
JOURNAL INFORMATION
==============================
NLM Title Abbreviation: Buffalo Med J Mon Rev Med Surg Sci
NLM Journal ID: 0004276

The Buffalo Medical Journal and Monthly Review of Medical and Surgical Science

Publisher: Biomedical Journal Digitization Project

ARTICLE INFORMATION
==============================
PMCID: PMC8936134
Article version: 1
Subjects: Editorial Department

Death of Prof. J. K. Mitchell

Print publication date: 1858 Jun
Volume: 14
Issue: 1
First page: 63
Last page: 63
License: This work is free of known copyright restrictions. For more information, please see PMC Back Issue Digitization.
License URL: https://creativecommons.org/publicdomain/mark/1.0/

==============================

sents a fine appearance, and contains many valuable articles. We welcome it to our exchange list.

We have also received the first number of the *Oglethorpe Medical and Surgical Journal*, issued bi-monthly, and edited by Drs. Byrd and Steele, of which we can also speak in no less complimentary terms.

---

*Death of Prof. J. K. Mitchell.*—We announce with deep regret, the death of Prof. J. K. Mitchell, who has long held the chair of Practice in the Jefferson Medical College, and who is known to the profession as one of the most eminent teachers of that branch we have ever had among us. Prof. Mitchell has suffered of late years from partial hemiplegia, but has been able to fulfill the duties of his chair up to the close of the last session, when his health was better than it had been for some years. He was attacked, however, this spring, with pneumonia, which terminated fatally, taking from us one of our profession's noblest ornaments. Dr. Mitchell was distinguished for his devotion to polite literature as well as the cause of medical science; he was an honorable, high-minded man, and a kind and courtly gentleman of the old school. The vacancy has been filled by the appointment of Dr. Dickson, the eminent Professor of Practice at Charleston, who was unanimously recommended by the faculty.

We learn also from the North American Medico-Chirurgical Review, of the death of Benjamin Travers, the distinguished English surgeon; Dr. Forbes Royle, the author of a well known work on Materia Medica; Dr. I. K. Matther, of the University of Leipzig; Dr. Schoepf, of the University of Pesth; and M. L. Bandon, of Paris. We have also notice in the daily papers an announcement of the death of M. Chomel.

---

*Books and Pamphlets Received.*—We have received from the publisher, Barclay on Medical Diagnosis, Graham's Elements of Inorganic Chemistry, Wilson's Plates of Diseases of the Skin, the Transactions of the New York State Medical Society, Illinois State Medical Society; and from the author, Paine's Institutes of Medicine.

Reviews of these works have been crowded out by the Transactions of the
